# Factors influencing effective acquisition of primary care patients for cancer early diagnostics

**DOI:** 10.1186/1897-4287-13-S2-A18

**Published:** 2015-11-26

**Authors:** Paweł Żuk, Artur Prusaczyk, Anna Kołak, Małgorzata Kalisz, Tomasz Włodarczyk

**Affiliations:** 1 Medical and Diagnostic Center, Siedlce, Poland

## 

Realization of prevention diagnostics reaches unsatisfactory level in Poland. Average coverage ratio for cytology is 22.14% and 42.77% for mammography. Analogical ratios for Mazovia region reach the level of 21.47% for cytology and 40.61% for mammography. At the same time Centrum Medyczno-Diagnostyczne (CMD) achieved significantly higher coverage for its primary care population i.e. 56.48% for cytology and 50.37% for mammography. The aim of the analysis presented below is to find significant factors influencing effective acquisition of primary care patients for cancer early diagnostics.

The first main factor is low efficiency of traditional invitations sent via traditional mail by regional coordination centers. The efficiency ratio reaches the level of 0.31% for cytology and 4.49% for mammography. On the basis of CMD's experience it can be seen that in order to achieve a better coverage in cancer diagnostics apart from only passive types of acquisition such as traditional mailing which result in maximum 20% effectiveness, active types of acquisition that result in 40% effectiveness should be implemented. What is more, MPs and local authorities should be involved in the process of acquisition (maximum 10% of effectiveness). Due to the fact that acquisition costs (average 56.8% of total costs) raise progressively for more active types of acquisition there should be a progressive growth of the revenue rate in line with the growth of coverage rate in each healthcare providers' population.

While developing acquisition system in CMD four main parts were identified: plan development (goals setting), delegation of tasks, implementation of supervision and motivation system, constant education: staff, patients. What is more CMD implemented wide range of operational, supervision and motivation tools that are of vital importance in the process of cancer prevention diagnostics acquisition, e.g. prevention diagnostics monitoring tool - that is used by medical receptionists, nurses and midwifes to manage acquisition of patients, realization of prevention diagnostics for each MP report - that is analyzed and distributed for MPs in CMD, calculation of prevention diagnostics realization ratio that influences MPs' bonuses.

Starting from the beginning of 2013 till June 2014 the number of CMD primary care cytologies reach 11 098 (132 positive results) i.e. 84 cytologies for one positive result, respective statistics for mammographies are 3 715 (19 positive results) i.e. 196 mammographies for one positive result.

Taking everything into consideration, the main factors influencing effective acquisition of primary care patients for cancer early diagnostics are: implementation of active types of acquisition, creation of a team and tasks delegation, monitoring and motivation system with several monitoring and motivation tools, real access to diagnostics, inclusion of prevention diagnostics into occupational medicine, access to PESEL and phone number database, support of local authorities and NGOs and education of staff and patients.

